# Safety evaluation of saffron stigma (*Crocus sativus* L.) aqueous extract and crocin in patients with schizophrenia

**Published:** 2015

**Authors:** Bentolhoda Mousavi, Seyedeh Zahra Bathaie, Farbod Fadai, Zabihollah Ashtari, Neda Ali beigi, Sara Farhang, Sara Hashempour, Nasim Shahhamzei, Hamid Heidarzadeh

**Affiliations:** 1 *Department of Psychiatry, University of Social Welfare and Rehabilitation Sciences, P.O.Box: 1985713834,Tehran, Iran.*; 2 *Department of Clinical Biochemistry, Faculty of Medical Sciences, Tarbiat Modares University, P.O.Box: 14115-111, Tehran, Iran.*; 3 *Clinical psychiatry research center, Tabriz University of Medical Sciences, Tabriz, Iran. *

**Keywords:** *Saffron Aqueous Extract*, *Crocin*, *Clinical trial*, *Safety assessment*, *Schizophrenia*

## Abstract

**Objectives:**

Saffron is the stigma of *Crocus sativus* L., which has the potentials to play a role in the treatment of many diseases. Although many researches are now going on this precious spice, there are few data on saffron safety in human, especially in patients with chronic mental illnesses. This study aimed to evaluate the short-term safety and tolerability of both saffron and crocin (its major constituent) in adult patients with schizophrenia**.**

**Materials and Methods:**

The capsules of saffron aqueous extract (SAE) and crocin were used to evaluate short-term safety and tolerability in patients with schizophrenia. A double-blind, placebo-controlled study was performed on patients with schizophrenia. The patients were all male and were divided into three 22-patient groups. While receiving their normal treatment, they also received a 12 week treatment with SAE (15 mg twice daily), crocin (15 mg twice daily) or placebo.

**Results:**

A total of 61 patients completed the trial; none of them reported a serious side effect. WBC count increased significantly in patients receiving saffron aqua extract (SAE), but it was within the normal range and had no clinical significance. Other hematologic components, markers of thyroid, liver and kidney or inflammation markers had no statistically significant difference among the groups.

**Conclusion:**

This study showed that SAE and crocin in doses of 15 mg twice daily were safely tolerated in patients with schizophrenia**.**

## Introduction

Saffron is the stigma of *Crocus sativus* L. and is a famous ancient herbal medicine. Recently, a wide range of beneficial effects have been reported for both saffron and its active constitute crocin, which has resulted in a growing interest among researchers. Reports have shown that saffron and crocin might have various pharmacological activities such as reducing the damage of ischemia/reperfusion (Hosseinzadeh et al. 2009 ▶), reducing markers of oxidative stress (Moallem et al. 2014 ▶), anticancer effect (Samarghandian et al. 2013 ▶), decreasing sexual side effect of serotonin pump inhibitors (Kashani et al. 2013 ▶), antinociceptive (Hosseinzadeh et al. 2002 ▶) as well as memory enhancing (Papandreou et al. 2011 ▶) and neuroprotective effects (Ghadrdoost et al. 2011 ▶). Saffron’s therapeutic effects have also been reported in disorders like depression (Hausenblas et al. 2013 ▶). Another report has shown its effect on lowering systolic blood pressure in rats (Imenshahidi et al. 2013 ▶). Saffron may also improve lipid profile and attenuate insulin resistance (Shirali et al. 2013 ▶). The results of these studies suggest this herbal remedy as a potential agent for add-on treatment for disorders influencing several systems of the body, such as mental disorders. Chronic mental disorders are among the most costly disorders worldwide. Patients with mental disorders often face several problems as comorbid conditions of their principal diagnosis (like depression in patients with schizophrenia or cognitive decline in depression (Mulholland et al. 2000 ▶) or side effect of medications (sexual dysfunction by serotonin pump inhibitors and metabolic syndrome by antipsychotics (Hert et al. 2009 ▶) .

In spite of many researches on this precious spice, more information is needed to assess its safety in patients with different diseases. Many studies have shown safety of saffron administration in animals; e.g., a study has shown that saffron is safe to be used orally in doses up to 5 g/Kg in rats (Ramadan et al. 2012 ▶). However, another study on Wistar rats has shown that intraperitoneal administration of saffron ethanolic extract can result in hepatic and renal tissue injuries (Mohajeri et al. 2007 ▶). Human studies have also evaluated safety of saffron and crocin; e.g. a study assessed administration of 20 mg crocin for one month in volunteers (Mohamadpour et al. 2013 ▶) and showed that crocin is relatively safe. Other studies have assessed safety of administration of 200 and 400 mg of saffron for one week in healthy volunteers and reported promising results (Modaghegh et al. 2008 ▶, Ayatollahi et al. 2013 ▶). However, there is still a need for more data on safety evaluation in human, especially in patients with chronic mental illnesses. This study aimed to evaluate short-term safety and tolerability of both saffron and crocin in adult patients with schizophrenia in a larger sample compared to previous reports. 

## Materials and Methods

The present study is a part of a larger trial on the beneficial effects of the add-on saffron extract for patients with schizophrenia, another part of this study has been recently published (Fadai et al. 2014). This trial has been registered with the Iranian Registry of clinical Trials (IRCT) (IRCT2013020312351N1) and was conducted in Razi Psychiatric Hospital, Tehran. The study was approved by ethical committee of University of Social Welfare and Rehabilitation Sciences, Tehran.

**Participants**

The participants were male adult inpatients (18 to 65 years old) who had fullfilled the DSM-IV-TR criteria for schizophrenia based on structured clinical interview which was carried out by a 3^rd^ year psychiatry resident. The patients were clinically stable and were receiving the same antipsychotic medication as their principal treatment. The purpose of this double-blind placebo controlled clinical trial was explained to the patients and they were included upon agreement and signing written informed consent. They were also informed that consent withdrawal would not influence their standard care.

Patients with acute general medical problems (including coronary heart diseases, metabolic syndromes, neurological diseases), substance related disorders, anti-platelets medications or using herbal medicines, allergy to saffron products, or intellectual disability were excluded. Patients were also excluded if they developed any moderate to severe side effects, change in antipsychotic treatment regimen based on the attending psychiatrist’s opinion and in case of consent withdrawal. 

**Study design**

Selected patients were randomized by computer generated codes and received either capsule of SAE (15 mg, two times a day) or capsule of crocin (15 mg, two times a day) or placebo (similar capsules filled with vehicle, two times a day). 

Side effects were systematically recorded throughout the study every other day during the first week, and then on weeks 2, 6 and 12. This assessment started with an open ended question and then continued using a checklist including hypersensitivity, abnormal bleeding, GI disturbances and general satisfaction (Modaghegh et al. 2008 ▶). 

*Saffron aqueous extract and crocin*

Saffron aqueous extract (SAE) and crocin were extracted and purified using the method described in our previous reports (Shirali et al. 2012 ▶, Shirali et al. 2013 ▶). Then, identical capsules were filled with crocin or SAE. Each capsule contained 15 mg dried SAE or 15 mg crocin, plus vehicle. Placebo capsules were also filled with vehicle.

Hematologic testing included complete blood count (CBC), liver function tests (serum Aspartate aminotransferase (AST), serum Alanine transaminase (ALT)), kidney function tests (urea, creatinine), endocrine evaluation tests (thyroid stimulating hormone, T4, T3) and biomarkers of inflammation (CRP, ESR) which were done at the beginning of the study. Testing was repeated in weeks 2, 6, and 12. Subjective reports of side effects were systematically recorded as explained earlier. 

**Statistical analysis**

The sample size was estimated 18 in each group according to Cohen Table considering a=0.05, β=0.2, S=5 and power=80%. Allowing for dropouts, 22 patients were recruited in each group.

Data are presented here as mean (Standard deviation). A chi-square test evaluated differences in the baseline characteristics and the presence of metabolic syndrome between the groups. A two-way repeated measures analysis of variance (time–treatment interaction) with a two-tailed Post Hoc LSD mean comparison test was performed. The type of medication was considered as a between-subjects factor (group) and time of measurements as the within-subjects factor (time). Differences were considered significant at P<0.05.

## Results

After evaluations, 66 patients with schizophrenia were considered eligible for participation. From them, 22 patients were randomly assigned to each group. Mean age (SD) of patients was 49.3 7.1 years in the SAE group, 48.17.7 years in the crocin group and 48.1 6.1 years in the placebo group (p=0.811). 

During the study, 2 patients were excluded from the SAE group (1 was diagnosed with cancer, 1 withdrew his consent), 2 from crocin group (1 for consent withdrawal and 1 for early discharge) and 1 from the placebo group (for consent withdrawal). Thus, a total of 20 patients completed the study in SAE group, 20 in crocin group and 21 in the placebo group. No serious adverse effect was reported by these patients.

Hematologic indices as measured at baseline and weeks 2, 6 and 12 are described in Table 1. A repeated measure analysis indicated that WBC increased significantly in patients receiving SAE compared to patients receiving crocin or placebo during the study period. However, this increase was within the normal range and had no clinical significance. Other indices did not have a significant change during the trial and were not different among the groups.

Markers of thyroid, liver and kidney function were also compared between these groups in baseline and week 12. As described in Table 2 and 3, none of these markers changed significantly during the study and there was no difference among the groups. 

As described in Table 4, level of inflammatory markers did not have a significant change during the study in neither of the groups, and was not different among the three groups.

**Table 1:** Effect of SAE, crocin and placebo on hematologic indices in patients with schizophrenia, as mean± SD

**Parameters Groups**		**Baseline **	**Week 2**	**Week 6**	**Week 12**	**p**
White blood cell count *10^3^	SAE	6.6±1.7	7.3±1.6	7.2 ±1.3	7.3 ±1.4	0.010
	Crocin	6.2 ±1.3	6.0 ±1.7	6.3±1.4	6.0±1.4	
	Placebo	6.0±1.1	5.7±1.0	6.2±1.3	6.1±1.5	
Red blood cell count *10^6^	SAE	4.9±0.5	4.9±0.6	5.0±0.5	5.1±0.5	0.855
	Crocin	4.8±0.4	4.8±0.5	5.0±0.5	5.0±0.6	
	Placebo	4.9±0.5	4.8±0.4	4.9±0.4	5.0±0.4	
Hemoglobin	SAE	14.4±1.1	14.4±1.0	14.1±1.2	14.2±1.2	0.480
	Crocin	14.8±1.4	14.3±1.3	14.5±1.0	14.3±1.1	
	Placebo	14.2±1.3	14.1±1.2	14.1±1.3	13.8±1.6	
Hematocrite	SAE	43.6±4.3	43.6±4.2	42.7±4.0	42.8±4.0	0.868
	Crocin	44.5±4.6	43.0±4.2	43.1±4.8	43.2±5.2	
	Placebo	43.5±4.4	43.2±4.1	42.6±3.8	42.0±4.2	
Platelet *10^3^	SAE	267.1±44.9	271.9±51.8	273.3±44.4	275.1±52.1	0.654
	Crocin	263.8±45.1	262.6±39.3	272.6±41.5	271.8±35.6	
	Placebo	258.2±34.9	244.7±37.5	270.0±44.1	270.3±44.5	

**Table 2:** Effect of SAE, crocin and placebo on liver and kidney functioning in patients with schizophrenia, as mean ±SD

**Parameters Groups**		**Baseline **	**Week 2**	**Week 6**	**Week 12**	**p**
AST	SAE	34.6±9.3	25.1±5.4	22.7±5.1	37.5±7.2	0.165
	Crocin	36.1±7.5	26.1±8.4	25.2±8.2	22.5±4.3	
	Placebo	29.6±8.9	22.1±8.2	20.5±5.2	19.6±5.6	
ALT	SAE	21.4±10.3	22.5±8.8	20.7±8.1	20.7±7.4	0.651
	Crocin	22.1±10.3	24.5±11.1	22.6±9.1	18.1±7.0	
	Placebo	18.9±8.4	21.0±9.6	19.7±6.4	19.9±7.3	
ALP	SAE	242.4±68.5	265.2±54.3	258.4±48.8	251.9±60.6	0.531
	Crocin	242.8±57.6	237.0±51.5	240.6±70.2	251.6±62.9	
	Placebo	250.3±55.6	232.9±47.2	219.9±66.2	242.2±61.1	
Urea	SAE	26.9±8.2	28.4±6.9	27.1±10.9	26.2±10.4	0.204
	Crocin	30.2±8..6	32.3±8.0	31.0±9.0	30.2±8.6	
	Placebo	28.1±6.8	30.9±6.7	31.5±6.8	32.6±12.6	
Creatinine	SAE	0.9±0.1	1.0±0.1	1.0±0.1	1.0±0.1	0.702
	Crocin	0.9±0.1	1.0±0.1	1.0±0.1	1.1±0.1	
	Placebo	1.0±0.1	1.0±0.1	1.0±0.1	1.1±0.1	

AST: Aspartate aminotransferase; ALT:Alanine aminotransferase; ALP: Alkaline phosphatase

**Table 3:** Effect of SAE, crocin and placebo on thyroid function in patients with schizophrenia, as mean ±SD

**Parameters Groups**		**Baseline **	**Week 2**	**Week 6**	**Week 12**	**p**
TSH	SAE	1.8±1.6	1.7±1.5	1.9±1.7	2.2±2.4	0.081
	Crocin	1.3±0.8	1.7±1.2	1.7±1.2	1.6±1.0	
	Placebo	1.1±0.6	1.2±0.6	1.1±0.4	1.0±0.5	
T3	SAE	0.7±0.1	0.8±0.1	0.8±0.1	0.8±0.1	0.449
	Crocin	0.7±0.1	0.8±0.1	0.8±0.1	0.8±0.1	
	Placebo	0.7±0.1	0.7±0.1	0.8±0.1	0.7±0.1	
	SAE	8.5±1. 8	8.5±1.8	8.6±1.7	8.5±1.8	
T4	Crocin	7.8±1.1	8.1±1.3	8.4±1.2	8.2±1.6	0.225
	Placebo	7.8±1.4	7.5±1.4	8.1±1.3	7.8±1.6	

TSH: Thyroid stimulating hormone; T3: triiodothyronine; T4: thyroxine

**Table 4:** Effect of SAE, crocin and placebo on markers of inflammation in patients with schizophrenia, as mean ±SD

**Parameters Groups**		**Baseline **	**Week 12**	**p**
ESR	SAE	7.0±4.3	6.1±2.9	0.919
	Crocin	7.0±7.1	7.2±8.1	
	Placebo	6.1±5.0	8.3±6.5	
CRP	SAE	0.2±0.7	0.1±0.2	0.856
	Crocin	0.1±0.2	0.1±0.4	
	Placebo	0.1±0.2	0.1±0.4	

ESR: Erythrocyte sedimentation rate; CRP: C-reactive protein.

## Discussion

The results of the present study showed that SAE and crocin were safely tolerated by patients with schizophrenia who were clinically stable using a second generation antipsychotic medication. SAE increased the WBC count. Indices of liver, kidney and thyroid function as well as inflammatory markers did not change during the study.

There are few studies evaluating safety of saffron and crocin in human and are mainly limited to healthy volunteers. A one week trial of 200 and 400 mg saffron stigma tablets consumed by 10 healthy volunteers showed that saffron might decrease RBC, hemoglobin, Hct and platelets within normal ranges without any clinical significance (Modaghegh et al. 2008 ▶). Another report did not find any significant change in WBC count except for a decrease in mixed percentage of WBC in patients receiving 20 mg crocin (Mohamadpour et al. 2013 ▶). Results of both studies were compatible with the present study in terms of liver, kidney and thyroid function tests as well as markers of inflammation. However, these studies differ from the current study in terms of the number of measurements, analyzing method and dose of coercion. 

Another group of studies evaluated the therapeutic effect of saffron (or its active components) in different clinical conditions. The results of these studies mostly indicated safety of using these products in patients with ischemic heart diseases, depression, mild to moderate Alzheimer’s disease or sexual dysfunction (Akhondzadeh et al. 2005 ▶, Akhondzadeh et al. 2010 ▶, Akhondzadeh et al. 2010 ▶, Cai et al. 2013 ▶, Shahmansouri et al. 2014 ▶).

According to our literature survey, there was no report about using saffron products in patients with mental illnesses, especially schizophrenia. Therefore, this study can be helpful in drawing a schema of using saffron in these patients. Beside the placebo controlled and double blinded design of this study, continual measurement of indices and larger number of subjects are among the strength points of this report. However, this study had some limitations as well. This study did not measure all components which could be potentially influenced by saffron (like blood coagulation time); however, we tried to select elements which are most susceptible to change according to previous reports. Thus, these results will have their clinical value when considering the limitations. 

In conclusion, this study found that SAE (30 mg daily) and crocin (30 mg daily) were safely tolerated by patients with schizophrenia during 12 weeks and no serious side effects were observed. SAE resulted in an increase in WBC count, which was not clinically significant. The results of this study can assist further research on the possible therapeutic effects of SAE and crocin in patients with schizophrenia.
